# Supplementary material for: Variations in Rates of Discharges to Nursing Homes after Acute Hospitalization for Stroke and the Influence of Service Heterogeneity: An Anglia Stroke Clinical Network Evaluation Study
Source: Healthcare (Basel). 2020 Oct 9;8(4):390. doi: 10.3390/healthcare8040390 (PMC7712187; doi:10.3390/healthcare8040390)
Supplement: Supplementary file 1 [file healthcare-08-00390-s001.pdf]

Supplementary Material

CONTENTS

List of Supplementary Tables

Supplementary Table S1 Summary of findings from a priori literature review evaluating what patient factors influence nursing home placement (institutionalization) after acute hospitalization for stroke

Supplementary Table S2 Distribution of patient risk factors of the 1335 patients included in analysis per individual hospital (n [%] unless otherwise stated).

Supplementary Table S3 Breakdown of the type of inpatient complications recorded and the proportion of patients recorded with each

Supplementary Table S4 Variables used to inform multiple imputation of missing data

Supplementary Table S5 Comparison of patient characteristics between complete cases and those with at least one missing variable

Supplementary Table S6 Multiple logistic regression complete case analysis for new nursing home placement after hospitalization for stroke (n=972)

List of Supplementary Figures

Supplementary Figure S1 Flow chart of cases included for the analysis, illustrating the reasons for exclusion.

Supplementary Figure S2 Stacked bar chart showing the proportion of patients that were discharged either to a nursing home (red), back home (blue), to an interim/rehabilitation setting

(grey) or died as an inpatient (white), per hospital. H1 to H8 represent hospitals 1 to 8 inclusively.

Supplementary Figure S3 Model estimates of new nursing home placement odds ratio for each hospital and hospital type. Horizontal line represents an odds ratio of 1 for reference hospital 1; its green colour represents that it is a tertiary hospital. Multivariable regression model was adjusted for pre-stroke mRS score, discharge mRS score, TACS, myocardial infarction or ischemic heart disease, complication, brain lateralization, and acute hospital length of stay, after multiple imputation for missing covariate data.

Supplementary Figure S4 Model estimates of new nursing home placement odds ratio for each hospital against size of hospital (represented as number of total hospital beds). Horizontal line represents an odds ratio of 1 for reference hospital 1. Vertical line represents the size of hospital 1. Multivariable regression model was adjusted for pre-stroke mRS score, discharge mRS score, TACS, myocardial infarction or ischemic heart disease, complication, brain lateralization, and acute hospital length of stay, after multiple imputation for missing covariate data.

Supplementary Figure S5 Model estimates of new nursing home placment odds ratio for each hospital against hospital stroke volume (mean number of stroke patients admitted and treated in each hospital per month). Horizontal line represents an odds ratio of 1 for reference hospital 1. Vertical line represents the stroke volume of hospital 1. Multivariable regression model was adjusted for pre-stroke mRS score, discharge mRS score, TACS, myocardial infarction or ischemic heart disease, complication, brain lateralization, and acute hospital length of stay, after multiple imputation for missing covariate data.

Supplementary Figure S6
Model estimates of new nursing home placement odds ratio for each hospital against distance to neurosurgical facilities (in miles). Horizontal line represents an odds ratio of 1 for reference hospital 1. Vertical line illustrates that neurosurgery was available onsite at hospital 1. Multivariable regression model was adjusted for pre-stroke mRS score, discharge mRS score, TACS, myocardial infarction or ischemic heart disease, complication, brain lateralization, and acute hospital length of stay, after multiple imputation for missing covariate data.

Supplementary Figure S7 Model estimates of new nursing home placement odds ratio for each hospital against presence of vascular surgery onsite. Horizontal line represents an odds ratio of 1 for reference hospital 1; its blue colour represents that it does not have a vascular surgery onsite. Multivariable regression model was adjusted for pre-stroke mRS score, discharge mRS score, TACS, myocardial infarction or ischemic heart disease, complication, brain lateralization, and acute hospital length of stay, after multiple imputation for missing covariate data.

Supplementary Figure S8 Model estimates of new nursing home placement odds ratio for each hospital against number of senior doctors per five stroke unit beds. Horizontal line represents an odds ratio of 1 for reference hospital 1. Vertical line represents the senior doctor staffing level for the hospital 1. Multivariable regression model was adjusted for pre-stroke mRS score, discharge mRS score, TACS, myocardial infarction or ischemic heart disease,

complication, brain lateralization, and acute hospital length of stay, after multiple imputation for missing covariate data.

Supplementary Figure S9 Model estimates of new nursing home placement odds ratio for each hospital against number of junior doctors per five stroke unit beds. Horizontal line represents an odds ratio of 1 for reference hospital 1. Vertical line represents the junior doctor staffing level for hospital 1. Multivariable regression model was adjusted for pre-stroke mRS score, discharge mRS score, TACS, myocardial infarction or ischemic heart disease, complication, brain lateralization, and acute hospital length of stay, after multiple imputation for missing covariate data.

Supplementary Figure S10 Model estimates of new nursing home placement odds ratio for each hospital against number of nurses per five stroke unit beds. Horizontal line represents an odds ratio of 1 for reference hospital 1. Vertical line represents the nurse staffing levels for hospital 1. Multivariable regression model was adjusted for pre-stroke mRS score, discharge mRS score, TACS, myocardial infarction or ischemic heart disease, complication, brain lateralization, and acute hospital length of stay, after multiple imputation for missing covariate data.

Supplementary Figure S11 Model estimates of new nursing home placement odds ratio for each hospital against number of occupational therapists per five bed days. Horizontal line represents an odds ratio of 1 for reference hospital 1. Veritical line represents the staffing levels of occupational therapists in hospital 1. Multivariable regression model was adjusted for pre-stroke mRS score, discharge mRS score, TACS, myocardial infarction or ischemic heart disease, complication, brain lateralization, and acute hospital length of stay, after multiple imputation for missing covariate data.

Supplementary Figure S12 Model estimates of new nursing home placement odds ratio for each hospital against the number of physiotherapists per five stroke unit beds. Horizontal line represents an odds ratio of 1 for reference hospital 1. Vertical line represents the staffing levels of physiotherapists in hospital 1. Multivariable regression model was adjusted for pre-stroke mRS score, discharge mRS score, TACS, myocardial infarction or ischemic heart disease, complication, brain lateralization, and acute hospital length of stay, after multiple imputation for missing covariate data.

Supplementary Figure S13 Model estimates of new nursing home placement odds ratio for each hospital against the number of speech and language therapists per five stroke unit beds. Horizontal line represents an odds ratio of 1 for reference hospital 1. Vertical line represents the staffing levels of speech and language therapists in hospital 1. Multivariable regression model was adjusted for pre-stroke mRS score, discharge mRS score, TACS, myocardial infarction or ischemic heart disease, complication, brain lateralization, and acute hospital length of stay, after multiple imputation for missing covariate data.

Supplementary Figure S14 Model estimates of new nursing home placement odds ratio for each hospital against the number of dieticians per five stroke unit beds. Horizontal line represents an odds ratio of 1 for reference hospital 1. Vertical line represents the staffing levels of dieticians in hospital 1. Multivariable regression model was adjusted for pre-stroke mRS score, discharge mRS score, TACS, myocardial infarction or ischemic heart disease,

complication, brain lateralization, and acute hospital length of stay, after multiple imputation for missing covariate data.

Supplementary Figure S15 Model estimates of new nursing home placement odds ratio for each hospital against number of hospital beds per CT scanner. Horizontal line represents an odds ratio of 1 for reference hospital 1. Vertical line represents the numer of hospital beds per CT scanner in hospital 1. Multivariable regression model was adjusted for pre-stroke mRS score, discharge mRS score, TACS, myocardial infarction or ischemic heart disease, complication, brain lateralization, and acute hospital length of stay, after multiple imputation for missing covariate data.

Supplementary Figure S16 Model estimates of new nursing home placement odds ratio for each hospital against number of stroke unit beds per 100 admissions. Horizontal line represents an odds ratio of 1 for reference hospital 1. Verical line represents the number of stroke unit beds for hospital 1. Multivariable regression model was adjusted for pre-stroke mRS score, discharge mRS score, TACS, myocardial infarction or ischemic heart disease, complication, brain lateralization, and acute hospital length of stay, after multiple imputation for missing covariate data.

Supplementary Figure S17 Model estimates of new nursing home placement odds ratio for each hospital against number of non-stroke patients treated daily on the stroke unit per five beds. Horizontal line represents an odds ratio of 1 for hospital 1. Verical line represents the number of non-stroke patients treated daily on the stroke unit of hospital 1. Multivariable regression model was adjusted for pre-stroke mRS score, discharge mRS score, TACS, myocardial infarction or ischemic heart disease, complication, brain lateralization, and acute hospital length of stay, after multiple imputation for missing covariate data.

Supplementary Figure S18 Model estimates of new nursing home placement odds ratio for each hospital against number of patients with stroke treated daily outside the stroke unit per five beds. Horizontal line represents an odds ratio of 1 for reference hospital 1. Verical line represents the number of patients with stroke treated outside the stroke unit per five beds for hospital 1. Multivariable regression model was adjusted for pre-stroke mRS score, discharge mRS score, TACS, myocardial infarction or ischemic heart disease, complication, brain lateralization, and acute hospital length of stay, after multiple imputation for missing covariate data.

Supplementary Figure S19 Model estimates of new nursing home placement odds ratio for each hospital against presence of early supported discharge (ESD) policy. Horizontal line represents an odds ratio of 1 for reference hospital 1; its blue colour represents that it does not have an ESD policy. Multivariable regression model was adjusted for pre-stroke mRS score, discharge mRS score, TACS, myocardial infarction or ischemic heart disease, complication, brain lateralization, and acute hospital length of stay, after multiple imputation for missing covariate data.

Supplementary Figure S20 Model estimates of new nursing home placement odds ratio for each hospital against mean Index of Multiple Deprivation (IMD) score of the counties in which the hospital services, with 95% confidence intervals. Horizontal line represents an odds ratio of 1 for reference hospital 1; its blue colour represents that hospital 1 is located in a county

with an IMD mean score of 13.89. Multivariable regression model was adjusted for pre-stroke mRS score, discharge mRS score, TACS, myocardial infarction or ischemic heart disease, complication, brain lateralization, and acute hospital length of stay, after multiple imputation for missing covariate data.

Supplementary Table S1 *Summary of findings from a priori literature review evaluating what patient factors influence nursing home placement (institutionalization) after acute hospitalization for stroke*

**Independent Variable**	**Association**	**Frequency of studies reporting association**	**Studies**
**Age**	Older age, more likely institutionalized	9/11	[1-8]
	No association	2/11	[9-10]
**Sex**	Female sex, more likely institutionalized	2/7	[4,11]
	No association	5/7	[4-7, 11]
**Caregiver availability**	Presence of caregiver before stroke, more likely institutionalized	1/1	[1]
	Living with others before stroke, less likely institutionalized	1/3	[12]
	Living with others before stroke, no association	2/3	[9-10]
	Marital status, no association	2/2	[7,10]
**Race/ethnicity**	White vs. non-whites, less likely institutionalized	1/3	[5]
	Blacks and Hispanics vs. white, less likely institutionalized	1/3	[13]
	No association	1/3	[10]
**Pathological stroke type**	Haemorrhagic stroke vs. ischaemic (or specific subtypes), more likely institutionalized	2/5	[1,8]
	No association	3/5	[9-10,14]
**Pathological cause of stroke**	Large artery atherosclerosis or cardio-embolism vs. small vessel occlusion, more likely institutionalized	1/1	[1]
**Anatomical site of stroke**	Total anterior circulation stroke, more likely institutionalized	2/2	[3,12]
**Stroke Severity**	Higher severity, more likely institutionalized	7/7	[1-2,6,8-10]
**Post-Stroke Functional Status**	Poor functional status, more likely institutionalized	2/3	[5-6]

Independent Variable	Association	Frequency of studies reporting association	Studies
	No association	1/3	[10]
	Cognitive impairment, more likely institutionalized	1/1	[7]
**Diabetes Mellitus**	Diabetes mellitus, more likely institutionalized	1/2	[1]
	No association	1/2	[7]
**Previous stroke/TIA**	Previous stroke, more likely institutionalized	2/2	[1,8]
	Previous TIA, no association	2/2	[7,12]
**Cardiovascular Disease**	Presence of cardiovascular disease, more likely institutionalized	1/4	[6]
	Presence of cardiovascular disease, less likely institutionalized	1/4	[1]
	No association	2/4	[7,10]
**Other comorbid conditions**	Hip fracture or dementia, more likely institutionalized	2/2	[8,10]
	Hypertension or depression or cancer or atrial fibrillation, no association	4/4	[7-8,10,12,]
**Complications**	Presence of complication, more likely institutionalized	2/3	[3,15]
	No association	1/3	[16]
**Other patient characteristics**	Longer AHLOS, more likely institutionalized	1/1	[7]
	Institutionalization prior to stroke, more likely institutionalized	1/2	[4,12]
	Pre-stroke accommodation, direction not reported	1/2	[4]
	Smoking, more likely institutionalized	1/2	[1]
	Smoking, less likely institutionalized	1/2	[6]

Independent Variable	Association	Frequency of studies reporting association	Studies
	Use of anticoagulants and antiplatelet drugs prior to stroke, more likely institutionalized	1/1	6
	Higher iScore, more likely institutionalized	1/1	17
	Socioeconomic status, educational attainment, BMI, functional status prior to stroke, aphasia, dysphagia, no association	5/5	6-7,9-10,18

AHLOS, acute hospital length of stay; BMI, body mass index; TIA, transient ischaemic attack.

List of References in Supplementary Table S1

1. Tseng HP, Lin FJ, Chen PT, et al. Derivation and validation of a discharge disposition predicting model after acute stroke. *J Stroke Cerebrovasc Dis*. 2015;24(6):1179-1186.
2. Mathisen SM, Larsen JP, Kurz MW. The prognosis of stroke survivors primarily discharged to their homes. *Acta Neurol Scand*. 2017;136(4):338-344.
3. Miu DK, Yeung JC. Incidence of post-stroke delirium and 1-year outcome. *Geriatr Gerontol Int.* 2013;13(1):123-129.
4. Rudd AG, Irwin P, Rutledge Z, Lowe D, Wade DT, Pearson M. Regional variations in stroke care in England, Wales and Northern Ireland: Results from the National Sentinel Audit of Stroke. Royal College of Physicians Intercollegiate Stroke Working Party. *Clin Rehabil*. 2001;15(5):562-572.
5. Schlegel DJ, Tanne D, Demchuk AM, Levine SR, Kasner SE. Prediction of hospital disposition after thrombolysis for acute ischemic stroke using the national institutes of health stroke scale. *Arch Neurol*. 2004;61(7):1061-1064.
6. Bejot, Yannick T, Odile G, et al. Poststroke disposition and associated factors in a population-based study: The Dijon Stroke Registry. *Stroke*. 2012;43(8):2071-2077.
7. Lai SM, Alter M, Friday G, Lai SL, Sobel E. Disposition after acute stroke: Who is not sent home from hospital? *Neuroepidemiology*. 1998;17(1):21-29.
8. Treger I, Ring H, Schwartz R, Tsabari R, Bornstein NM, Tanne,D. Hospital disposition after stroke in a national survey of acute cerebrovascular diseases in Israel. *Arch Phys Med Rehabil*. 2008;89(3):435-440.
9. Schlegel D, Kolb SJ, Luciano JM, et al. Utility of the NIH stroke scale as a predictor of hospital disposition. *Stroke*. 2003;34(1):134-137.
10. Bell CL, LaCroix AZ, Desai M, et al. Factors associated with nursing home admission after stroke in older women. *J Stroke Cerebrovasc Dis*. 2015;24(10):2329-2337.
11. Kapral MK, Fang J, Hill MD, et al. Sex differences in stroke care and outcomes: Results from the registry of the Canadian stroke network. *Stroke*. 2005;36(4):809-814.
12. Di Carlo A, Lamassa M, Baldereschi M, et al. Risk factors and outcome of subtypes of ischemic stroke. data from a multicenter multinational hospital-based registry. The European Community Stroke Project. *J Neurol Sci*. 2006;244(1-2):143-150.
13. Kind AJ, Smith MA, Liou JI, Pandhi N, Frytak JR, Finch MD. Discharge destination's effect on bounce-back risk in Black, White, and Hispanic acute ischemic stroke patients. *Arch Phys Med Rehabil*. 2010;91(2):189-195.

14. Barber M, Roditi G, Stott DJ, Langhorne P. Poor outcome in primary intracerebral haemorrhage: Results of a matched comparison. *Postgrad Med J*. 2004;80(940):89-92. doi: 10.1136/pmj.2003.010967.
15. Kwan J, Hand P. Infection after acute stroke is associated with poor short-term outcome. *Acta Neurol Scand*. 2007;115(5):331-338.
16. Finlayson O, Kapral M, Hall R, et al. Risk factors, inpatient care, and outcomes of pneumonia after ischemic stroke. *Neurology*. 2011;77(14):1338-1345.
17. Saposnik G, Raptis S, Kapral MK, et al. The iScore predicts poor functional outcomes early after hospitalization for an acute ischemic stroke. *Stroke*. 2011;42(12):3421-3428.
18. Pedersen PM, Jorgensen HS, Nakayama H, Raaschou HO, Olsen TS. Orientation in the acute and chronic stroke patient: Impact on ADL and social activities. The Copenhagen Stroke Study. *Arch Phys Med Rehabi*. 1996;77(4):336-339. doi: 10.1016/S0003-9993(96)90080-5.

Supplementary Table S2 *Distribution of patient risk factors of the 1335 patients included in analysis per individual hospital (n [%] unless otherwise stated).*

**Variables**	**Hospital 1 211 (16)**	**Hospital 2 10 (1)**	**Hospital 3 202 (15)**	**Hospital 4 63 (5)**	**Hospital 5 328 (25)**	**Hospital 6 168 (13)**	**Hospital 7 183 (14)**	**Hospital 8 170 (13)**	***P***
Age, y, median (IQR)*	76 (65 to 83)	83 (80 to 90)	78 (68 to 84)	74 (69 to 82)	76 (67 to 83)	75 (66 to 82)	78 (65 to 85)	78 (67 to 86)	0.09
Sex, female†	97 (46)	5 (50)	107 (53)	32 (51)	143 (44)	76 (45)	83 (45)	91 (54)	0.33
Haemorrhagic stroke†	20 (10)	0 (0)	20 (10)	4 (8)	26 (8)	16 (10)	17 (9)	9 (5)	0.70
Pre-stroke mRS ≥3†	7 (6)	2 (20)	30 (16)	-	35 (11)	12 (9)	11 (6)	18 (11)	0.04
Discharge mRS ≥ 3†	37 (24)	7 (70)	79 (42)	-	68 (22)	44 (37)	52 (30)	77 (45)	<0.001
TACS†	9 (5)	0 (0)	25 (13)	5 (11)	13 (4)	11 (7)	27 (17)	19 (11)	<0.001
No brain lateralization†	34 (17)	2 (20)	11 (6)	5 (8)	78 (24)	1 (1)	18 (10)	7 (4)	<0.001
Diabetes mellitus†	28 (13)	1 (10)	33 (16)	7 (11)	51 (16)	38 (23)	33 (18)	30 (18)	0.29
Myocardial infarction/ ischemic heart disease†	30 (14)	1 (10)	54 (27)	15 (24)	68 (21)	45 (27)	29 (16)	56 (33)	<0.001
Previous stroke †	31 (15)	1 (10)	32 (16)	8 (13)	68 (21)	35 (21)	44 (24)	32 (19)	0.18
Dementia†	13 (6)	1 (10)	13 (6)	0 (0)	17 (5)	10 (6)	11 (6)	8 (5)	0.66
Prior use of anti-coagulants or anti-platelets†	76 (44)	6 (60)	94 (53)	33 (52)	151 (48)	88 (52)	92 (51)	81 (48)	0.69
Complication†	49 (23)	3 (30)	20 (10)	19 (30)	92 (28)	50 (30)	43 (23)	31 (18)	<0.001
AHLOS, days, median (IQR)*	7 (4 to 16)	30 (17 to 39)	8 (5 to 22)	10 (4 to 27)	6 (4 to 11)	9 (5 to 21)	11 (6 to 23)	7 (3 to 20)	<0.001

AHLOS, acute hospital length of stay; IQR, interquartile range; mRS, modified Rankin Scale; TACS, total anterior circulation syndrome; y, years.

** Kruskal-Wallis rank sum test*

† *$\mathcal{X}^2$ test*

Supplementary Table S3 *Breakdown of the type of inpatient complications recorded and the proportion of patients recorded with each*

Complication Type	Number of patients	Percentage of total study population (n=1335)	Percentage of patients with complication recorded (n=307)
Another stroke	7	0.5	2
Pneumonia	67	5	22
UTI	96	7	31
Seizures	21	2	7
New MI	7	0.5	2
Acute coronary	7	0.5	2
Other (unspecified)	102	8	33

Supplementary Table S4 *Variables used to inform multiple imputation of missing data*

Variable	Measure	Missing (%)
I. Independent Variable		
Trust	0=Trust 1 1=Trust 2 2=Trust 3 3=Trust 4 4=Trust 5 5=Trust 6 6=Trust 7 7=Trust 8	-
II. Dependent Variable		
New Nursing Home Placement	0=No 1=Yes	-
III. Patient Risk Factors (Confounders)		
Pre-morbid mRS	0=scores 0 to 2 1= scores 3 to 5	219 (16)
Discharge mRS	0=scores 0 to 2 1= scores 3 to 5	206 (15)
TACS	0=No 1=Yes	105 (8)
Brain Lateralization	0=No 1=Yes	60 (4)
MI/IHD	0=No 1=Yes	-
Complication	0=No 1=Yes	-
AHLOS	Continuous, days	3 (0.2)
IV. Auxiliary Variables		
Sex	0=Male 1=Female	2 (< 1)
Age	Continuous, years	1 (< 1)
Previous Stroke	0=No 1=Yes	0 (0)
Diabetes Mellitus	0=No 1=Yes	0 (0)
Season of Admission	0=Summer 1=Winter	2 (< 1)
Day of Admission	0=Weekday 1=Weekend	2 (< 1)
ICU Admission	0=No 1=Yes	68 (5)
Dementia	0=No 1=Yes	0 (0)
Hypercholesterolemia	0=No 1=Yes	0 (0)
Myocardial Infarction or Ischemic Heart Disease	0=No 1=Yes	0 (0)
Hypertensive	0=No 1=Yes	0 (0)
Chronic Obstructive Pulmonary Disease	0=No 1=Yes	0 (0)
Stroke Type	0=Ischemic 1=Hemorrhagic	39 (2)
Atrial Fibrillation	0=No 1=Yes	172 (13)
Heart Rate	Continuous, beats per minute	143 (11)
Temperature	Continuous, ˚C	185 (14)
Use of anti-coagulants and/ antiplatelets prior to stroke	0=No 1=Yes	91 (7)

mRS, modified Rankin Scale; TACS, total anterior circulation stroke; myocardial infarction/ischemic heart disease; AHLOS, acute hospital length of stay; ICU, intensive care unit.

Supplementary Table S5 *Comparison of patient characteristics between complete cases and those with at least one missing variable*

Patient Characteristic	Complete Cases (n=972)	Cases with at least one missing variable (n=363)	*P*
	Median (IQR) or No. (%)		
Age, y*	77 (67 to 84)	75 (65 to 83)	0.04
Sex, female†	473 (49)	161 (44)	0.19
Comorbidities†			
Atrial Fibrillation	238 (28)	91 (28)	0.99
Previous Stroke	193 (20)	58 (16)	0.12
Diabetes Mellitus	164 (17)	57 (16)	0.67
Dementia	55 (6)	18 (5)	0.72
Hypercholesterolemia	192 (20)	58 (16)	0.14
Hypertensive	697 (72)	231 (64)	0.005
Myocardial Infarction or Ischemic Heart Disease	225 (23)	73 (20)	0.27
COPD	56 (6)	24 (7)	0.65
Pre-stroke mRS > 3	103 (11)	12 (8)	0.49
ICU admission	21 (2)	11 (3)	0.38
Hemorrhagic Stroke†	61 (6)	51 (15)	<0.001
TACS	92 (9)	17 (7)	0.19
No Brain Lateralization†	119 (12)	37 (12)	1
Complications	202 (21)	105 (29)	0.002
Discharge mRS Score > 3 ‡	316 (33)	48 (31)	0.70
Winter Admission†	512 (53)	197 (54)	0.61
Weekend Admission†	247 (25)	96 (27)	0.73
AHLOS, days	7 (4 to 17)	8 (4 to 20.25)	0.26
New Nursing Home Placement at discharge	105 (11)	30 (8)	0.21
Use of anticoagulants or antiplatelets before	464 (50)	157 (48)	0.63

IQR, interquartile range; COPD, chronic obstructive pulmonary disease; mRS, modified Rankin Scale; ICU, intensive care unit; TACS, total anterior circulation stroke.

Supplementary Table S6 *Multiple logistic regression complete case analysis for new nursing home placement after hospitalization for stroke (n=972)*

Independent Variable	OR	95% CI	*P*
Pre-stroke mRS (reference < 3)			
More than or equal to 3	0.77	0.35 to 1.69	0.51
Discharge mRS (reference < 3)			
More than or equal to 3	10.05	3.85 to 26.19	<0.001
TACS	0.94	0.41 to 2.14	0.87
MI/IHD	0.50	0.23 to 1.09	0.08
Had a complication	2.29	1.10 to 4.76	0.03
No brain lateralization	1.59	0.51 to 5.02	0.43
AHLOS, days	1.08	1.06 to 1.10	<0.001
Hospital (reference 1)			
2	0.42	0.03 to 5.78	0.52
3	5.54	1.16 to 26.51	0.03
5	1.75	0.36 to 8.53	0.49
6	0.31	0.05 to 2.01	0.22
7	1.08	0.22 to 5.34	0.92
8	0.56	0.11 to 2.82	0.48

OR, odds ratio; CI, confidence intervals; mRS, modified Rankin Scale; TACS, total anterior circulation stroke; MI/IHD, myocardial infarction or ischemic heart disease; AHLOS, acute hospital lengths of stay.

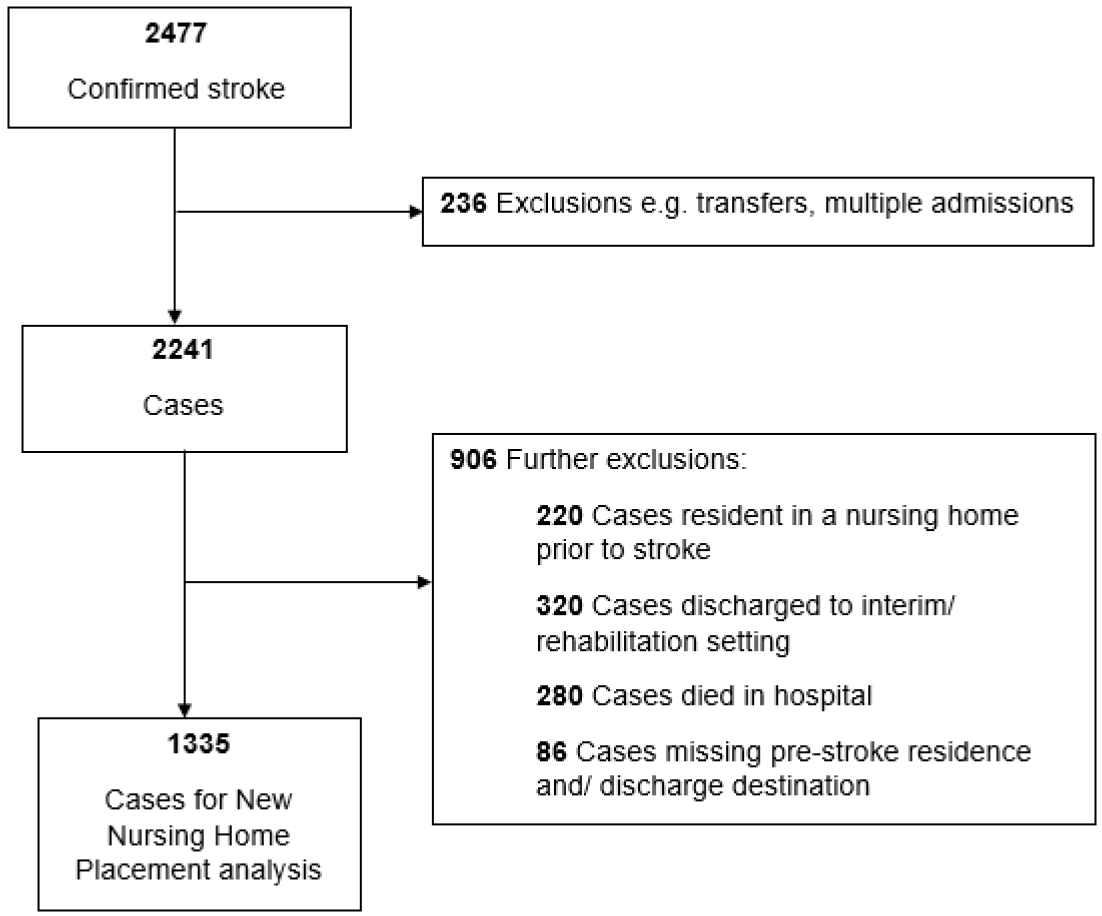

Supplementary Figure S1 *Flow chart of cases included for the analysis, illustrating the reasons for exclusion.*

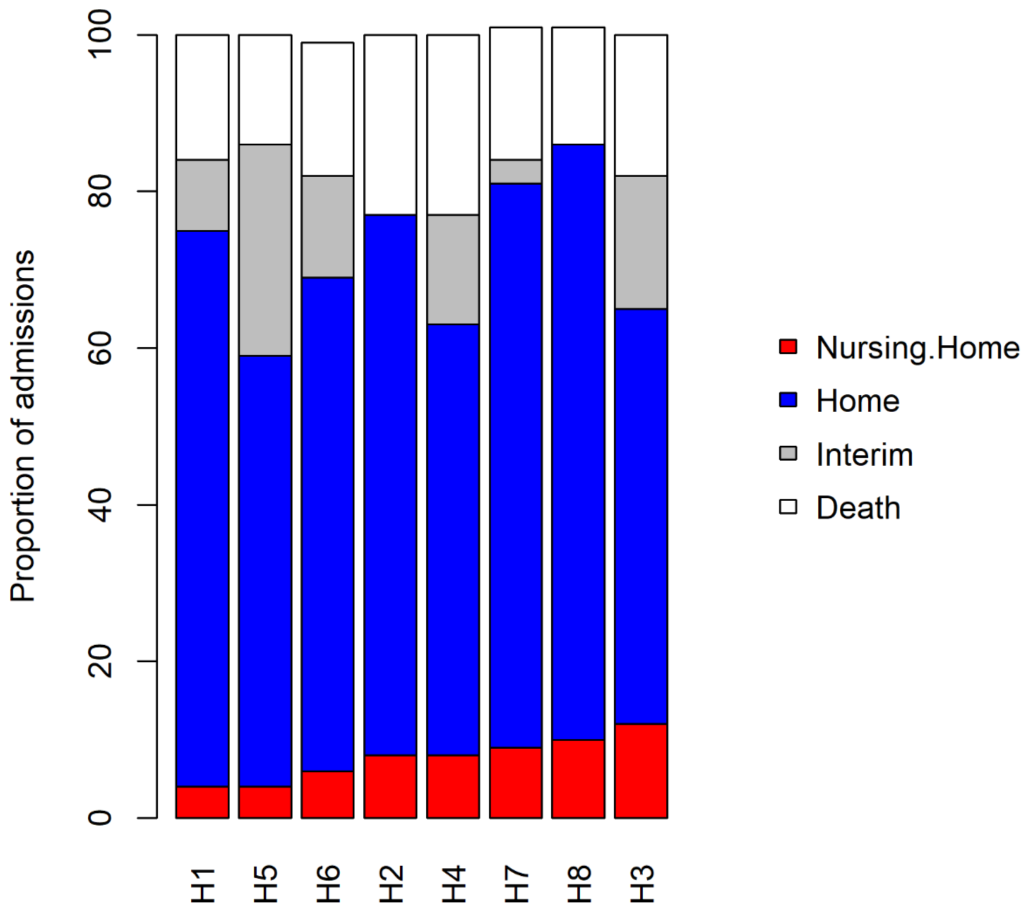

Supplementary Figure S2 *Stacked bar chart showing the proportion of patients that were discharged either to a nursing home (red), back home (blue), to an interim/rehabilitation setting (grey) or died as an inpatient (white), per hospital. H1 to H8 represent hospitals 1 to 8 inclusively.*

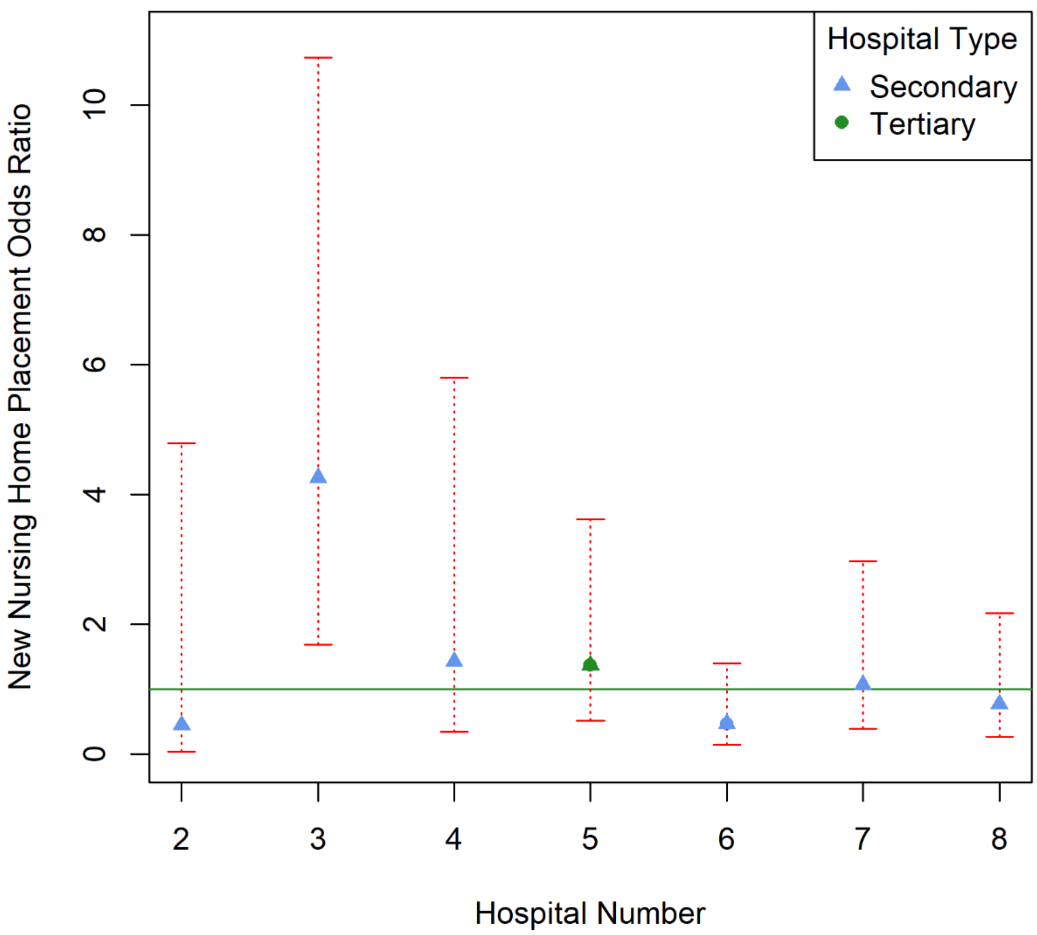

Supplementary Figure S3 *Model estimates of new nursing home placement odds ratio for each hospital and hospital type. Horizontal line represents an odds ratio of 1 for reference hospital 1; its green colour represents that it is a tertiary hospital. Multivariable regression model was adjusted for pre-stroke mRS score, discharge mRS score, TACS, myocardial infarction or ischemic heart disease, complication, brain lateralization, and acute hospital length of stay, after multiple imputation for missing covariate data.*

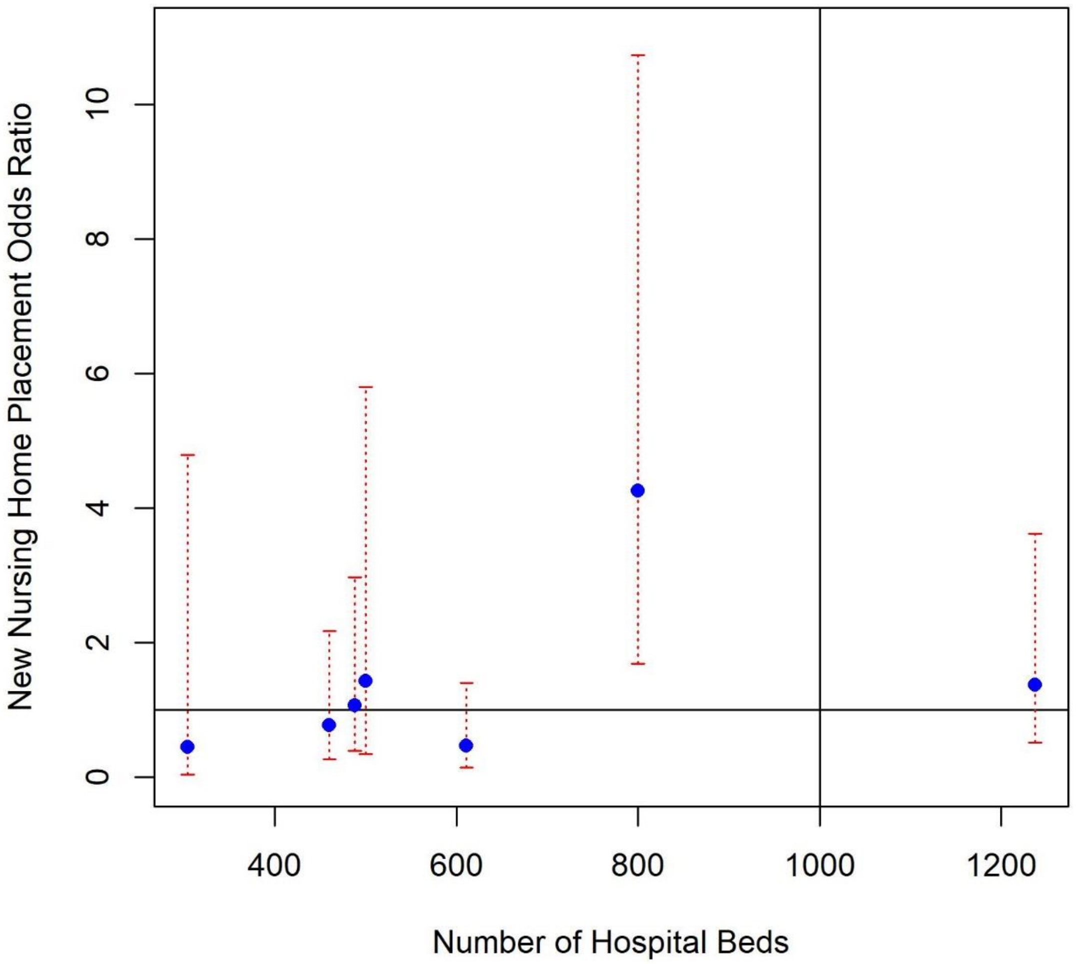

Supplementary Figure S4 *Model estimates of new nursing home placement odds ratio for each hospital against size of hospital (represented as number of total hospital beds). Horizontal line represents an odds ratio of 1 for reference hospital 1. Vertical line represents the size of hospital 1. Multivariable regression model was adjusted for pre-stroke mRS score, discharge mRS score, TACS, myocardial infarction or ischemic heart disease, complication, brain lateralization, and acute hospital length of stay, after multiple imputation for missing covariate data.*

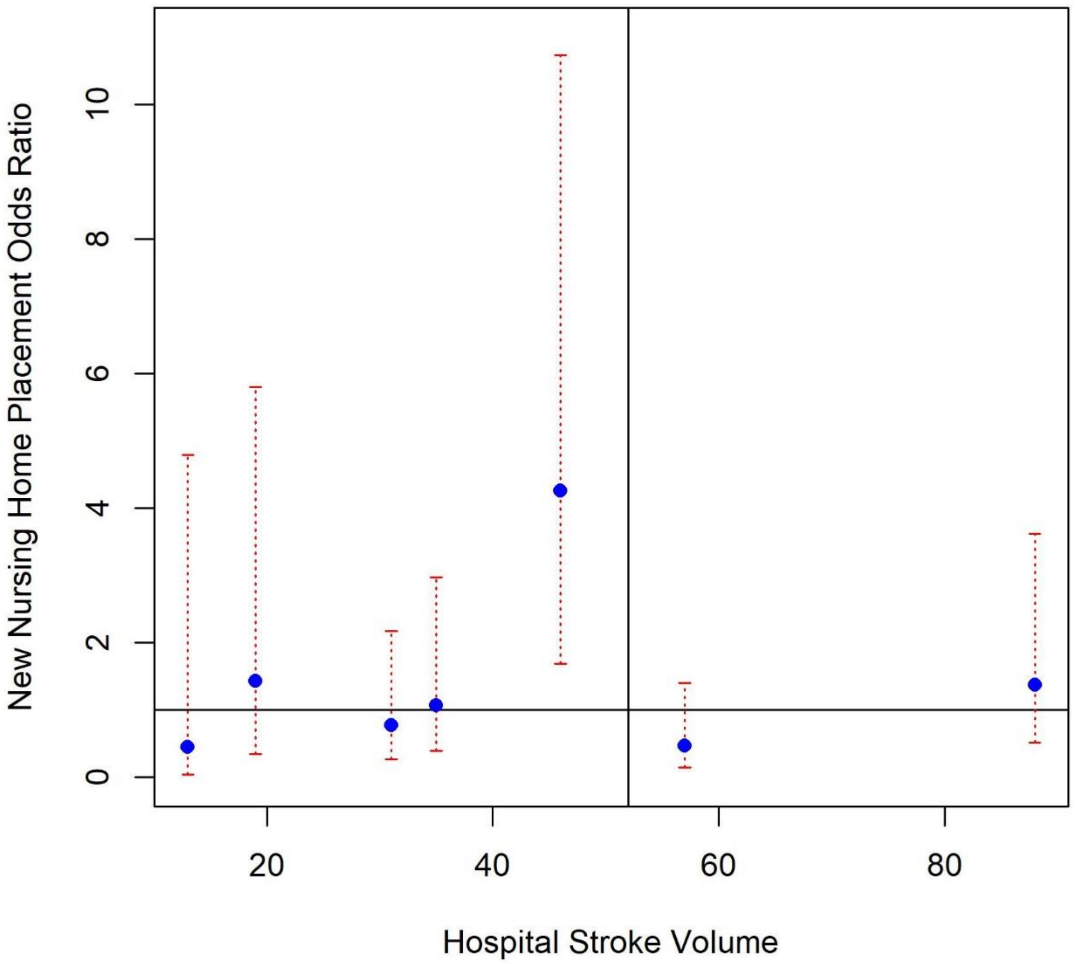

Supplementary Figure S5 *Model estimates of new nursing home placment odds ratio for each hospital against hospital stroke volume (mean number of stroke patients admitted and treated in each hospital per month). Horizontal line represents an odds ratio of 1 for reference hospital 1. Vertical line represents the stroke volume of hospital 1. Multivariable regression model was adjusted for pre-stroke mRS score, discharge mRS score, TACS, myocardial infarction or ischemic heart disease, complication, brain lateralization, and acute hospital length of stay, after multiple imputation for missing covariate data.*

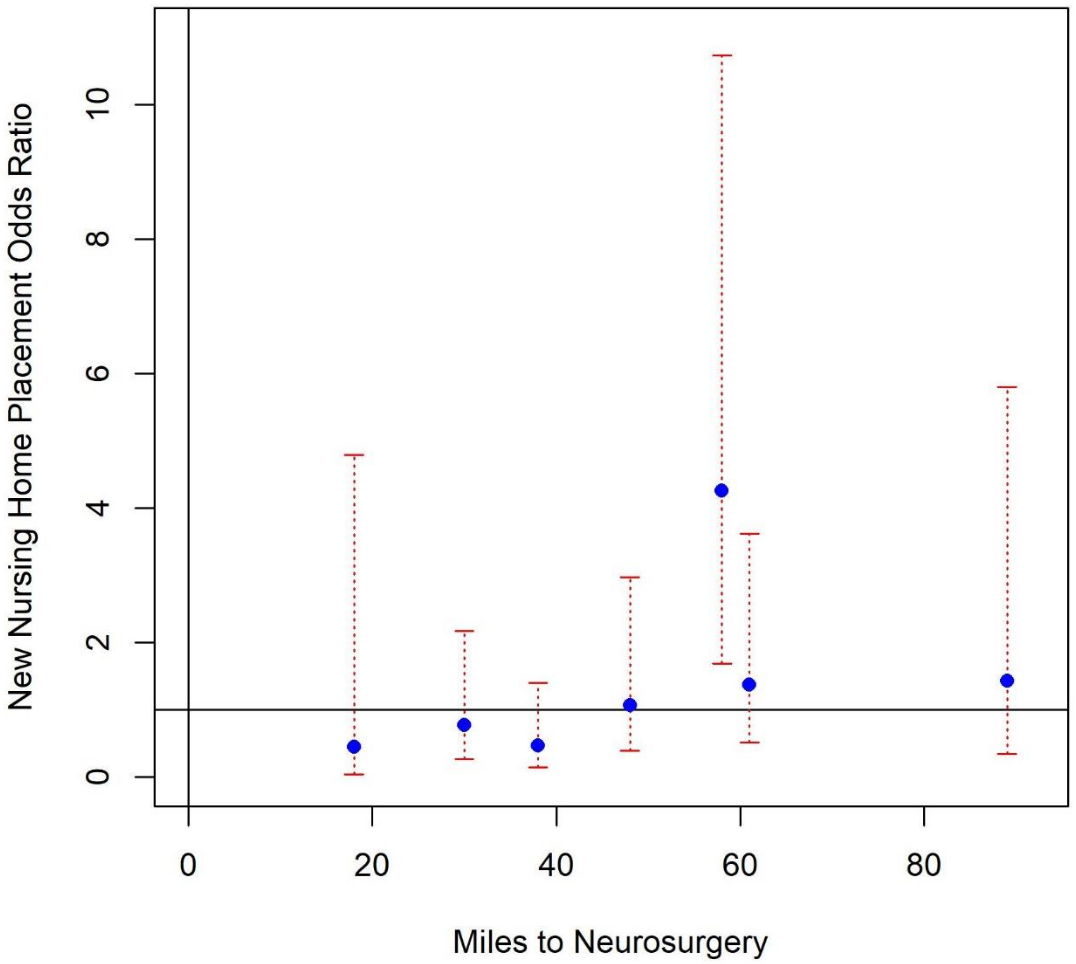

Supplementary Figure S6 *Model estimates of new nursing home placement odds ratio for each hospital against distance to neurosurgical facilities (in miles). Horizontal line represents an odds ratio of 1 for reference hospital 1. Vertical line illustrates that neurosurgery was available onsite at hospital 1. Multivariable regression model was adjusted for pre-stroke mRS score, discharge mRS score, TACS, myocardial infarction or ischemic heart disease, complication, brain lateralization, and acute hospital length of stay, after multiple imputation for missing covariate data.*

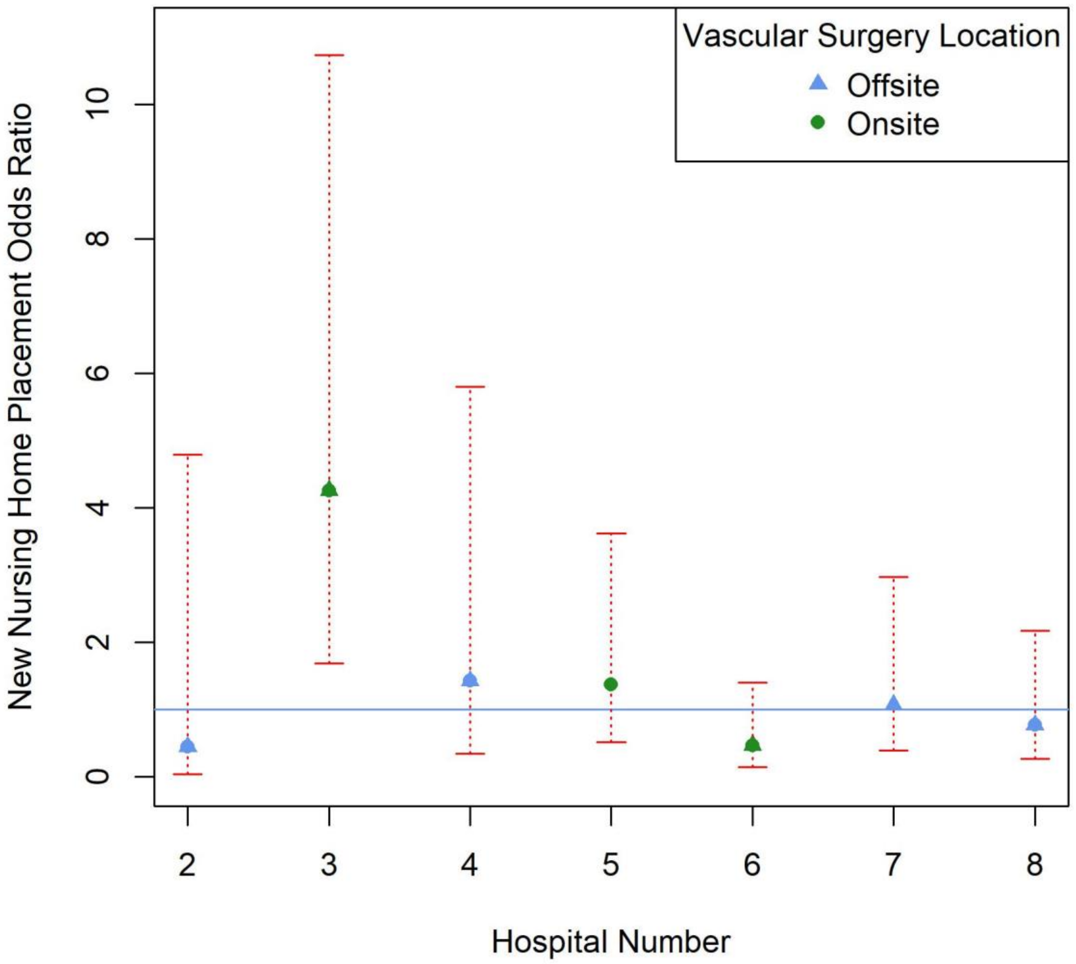

Supplementary Figure S7 *Model estimates of new nursing home placement odds ratio for each hospital against presence of vascular surgery onsite. Horizontal line represents an odds ratio of 1 for reference hospital 1; its blue colour represents that it does not have a vascular surgery onsite. Multivariable regression model was adjusted for pre-stroke mRS score, discharge mRS score, TACS, myocardial infarction or ischemic heart disease, complication, brain lateralization, and acute hospital length of stay, after multiple imputation for missing covariate data.*

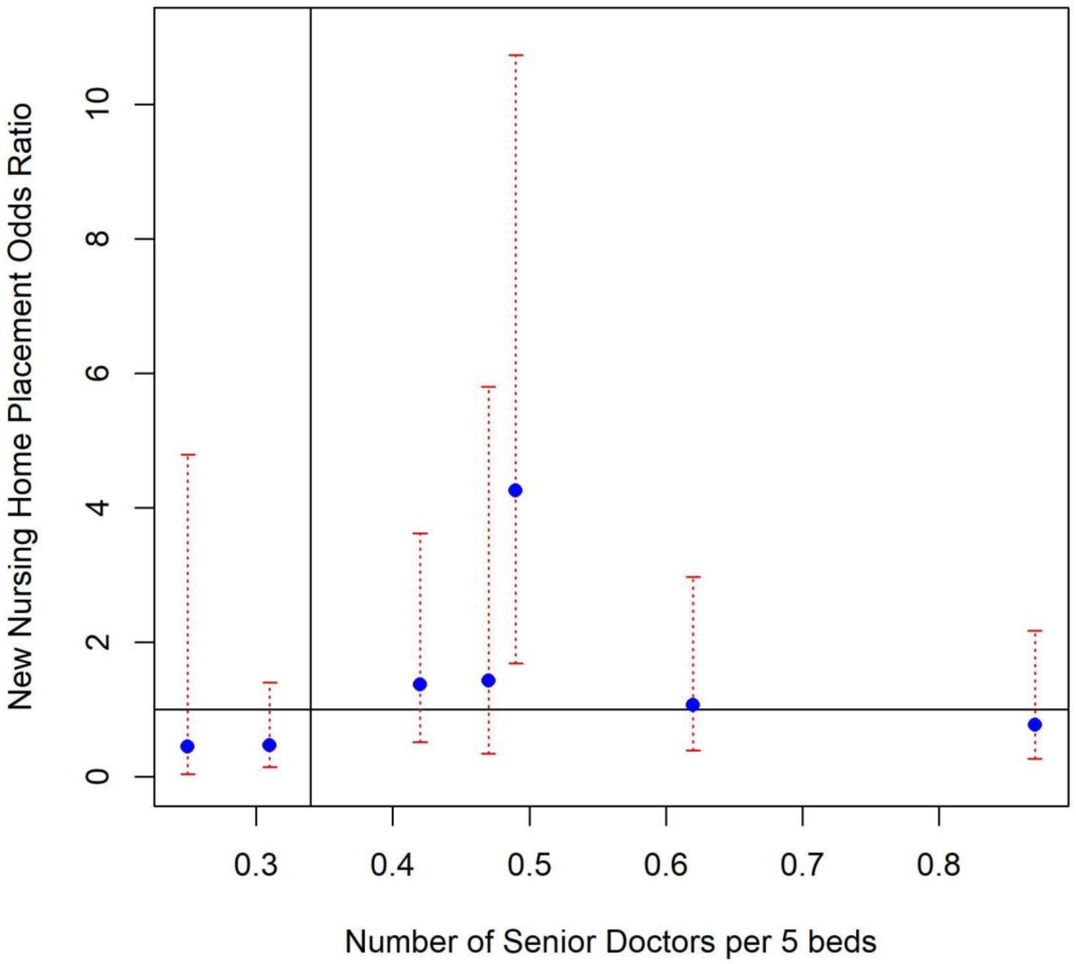

Supplementary Figure S8 *Model estimates of new nursing home placement odds ratio for each hospital against number of senior doctors per five stroke unit beds. Horizontal line represents an odds ratio of 1 for reference hospital 1. Vertical line represents the senior doctor staffing level for the hospital 1. Multivariable regression model was adjusted for pre-stroke mRS score, discharge mRS score, TACS, myocardial infarction or ischemic heart disease, complication, brain lateralization, and acute hospital length of stay, after multiple imputation for missing covariate data.*

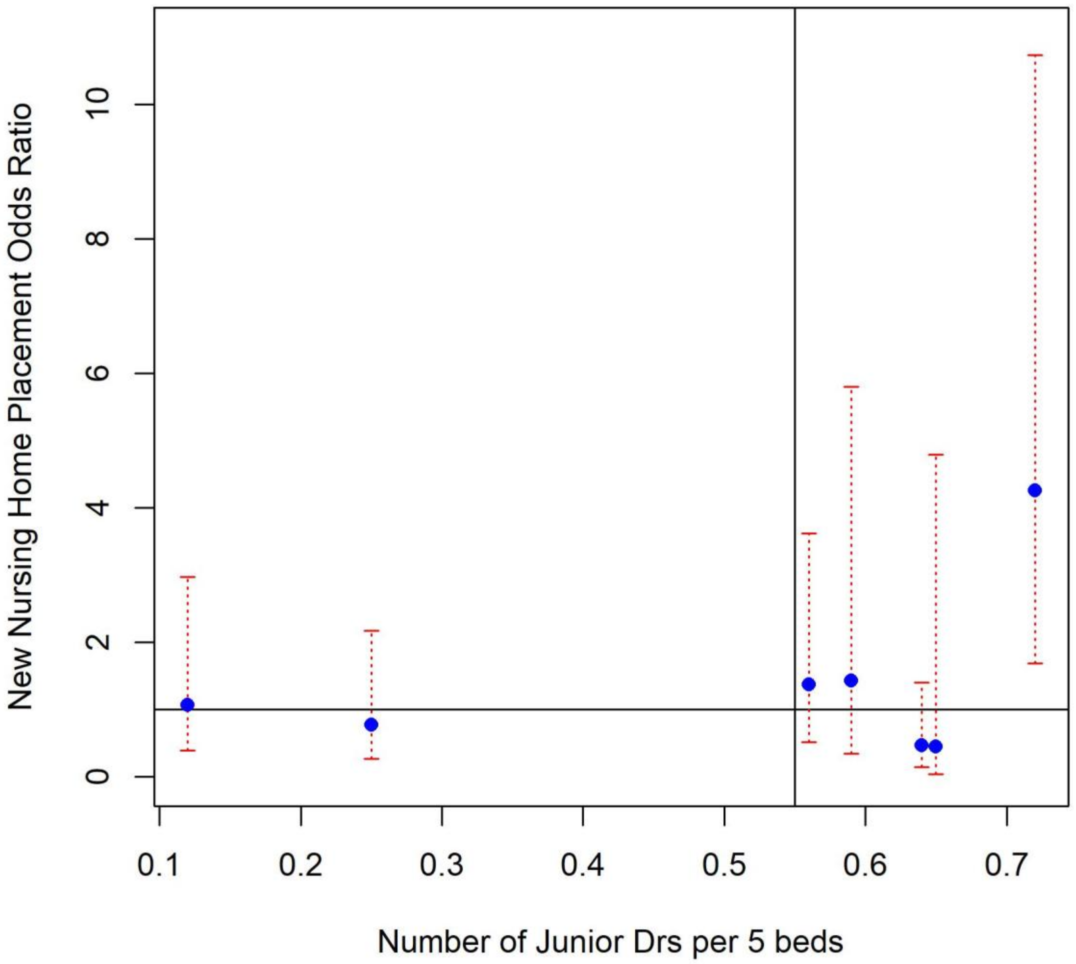

Supplementary Figure S9 *Model estimates of new nursing home placement odds ratio for each hospital against number of junior doctors per five stroke unit beds. Horizontal line represents an odds ratio of 1 for reference hospital 1. Vertical line represents the junior doctor staffing level for hospital 1. Multivariable regression model was adjusted for pre-stroke mRS score, discharge mRS score, TACS, myocardial infarction or ischemic heart disease, complication, brain lateralization, and acute hospital length of stay, after multiple imputation for missing covariate data.*

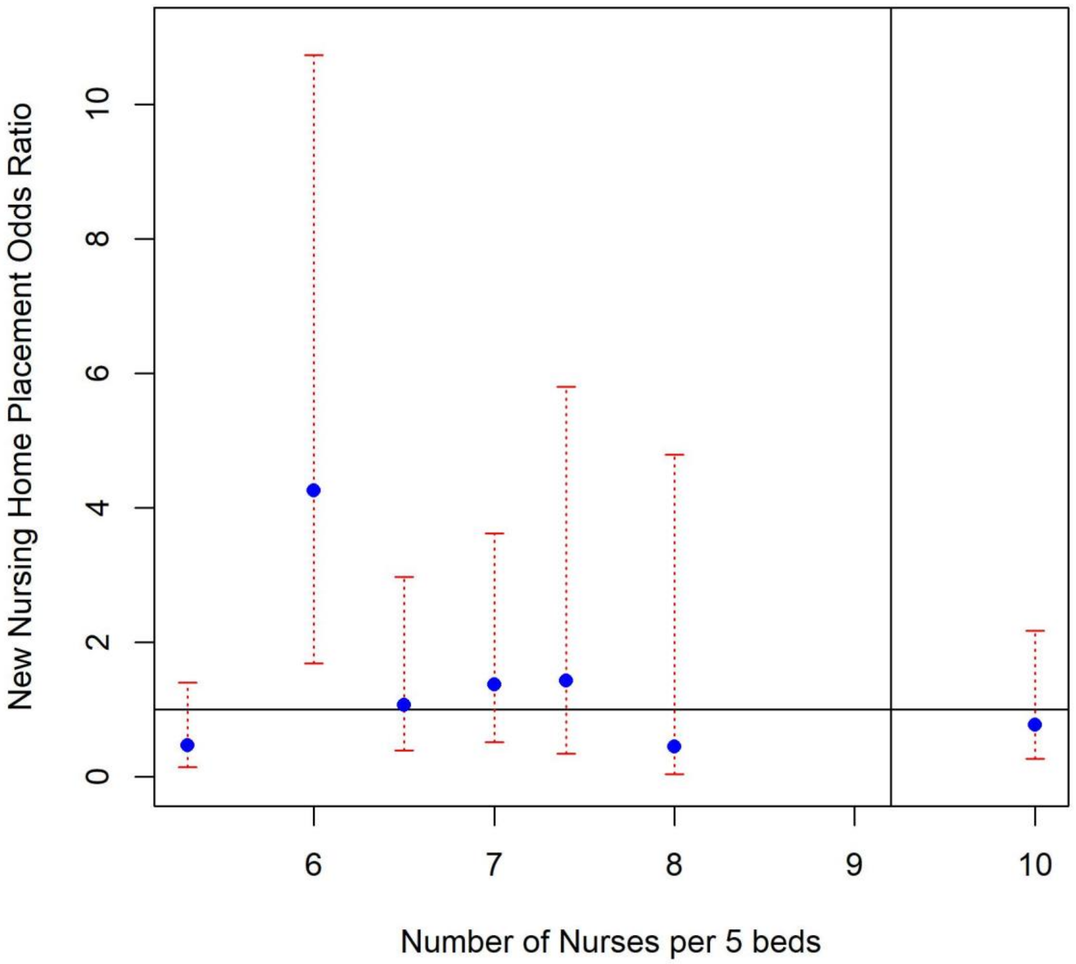

Supplementary Figure S10 *Model estimates of new nursing home placement odds ratio for each hospital against number of nurses per five stroke unit beds. Horizontal line represents an odds ratio of 1 for reference hospital 1. Vertical line represents the nurse staffing levels for hospital 1. Multivariable regression model was adjusted for pre-stroke mRS score, discharge mRS score, TACS, myocardial infarction or ischemic heart disease, complication, brain lateralization, and acute hospital length of stay, after multiple imputation for missing covariate data.*

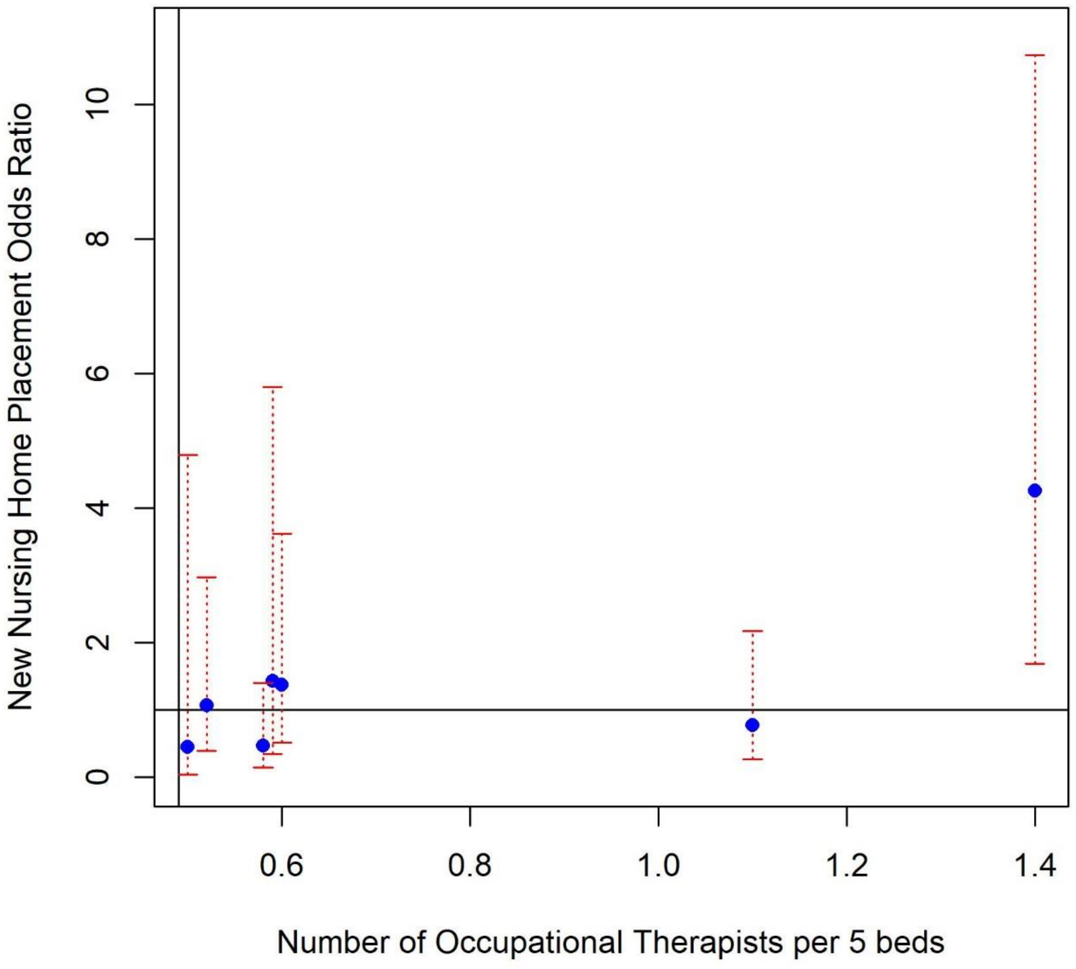

Supplementary Figure S11 *Model estimates of new nursing home placement odds ratio for each hospital against number of occupational therapists per five bed days. Horizontal line represents an odds ratio of 1 for reference hospital 1. Veritical line represents the staffing levels of occupational therapists in hospital 1. Multivariable regression model was adjusted for pre-stroke mRS score, discharge mRS score, TACS, myocardial infarction or ischemic heart disease, complication, brain lateralization, and acute hospital length of stay, after multiple imputation for missing covariate data.*

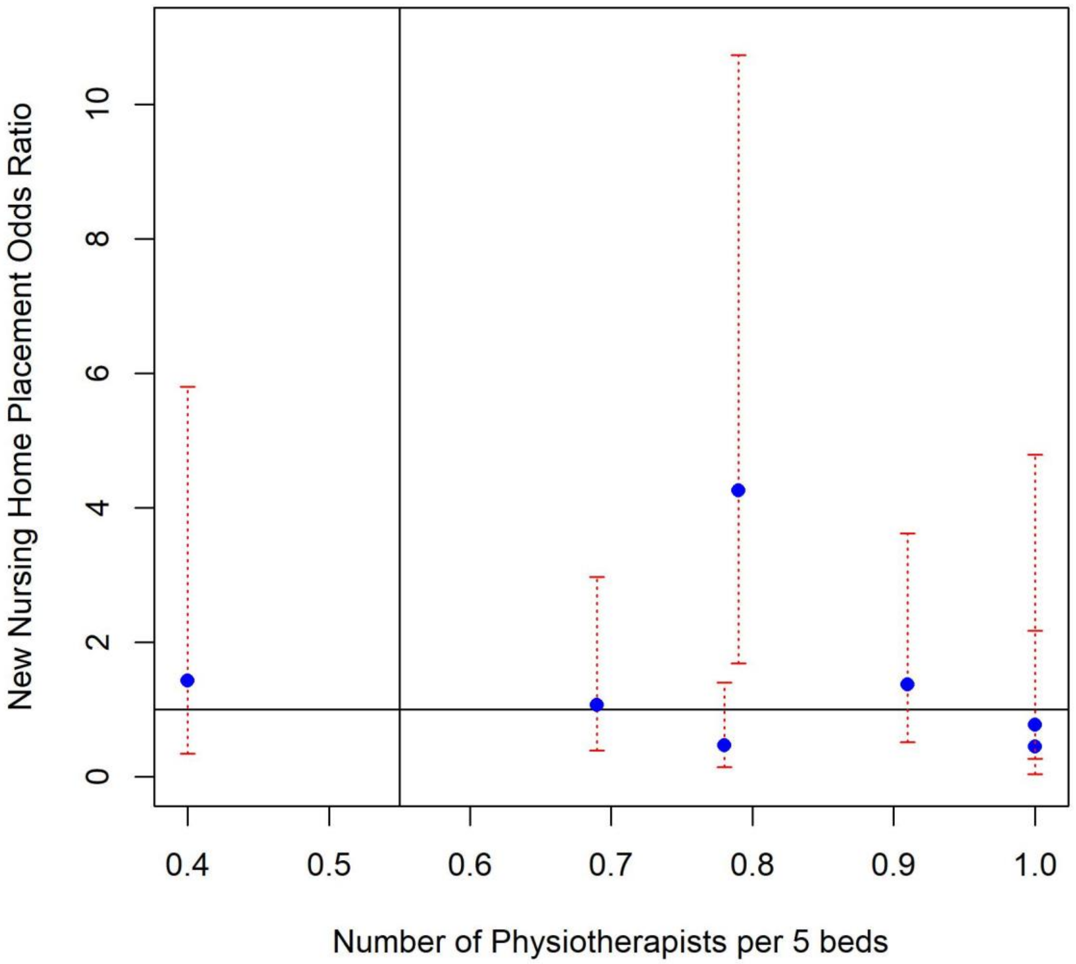

Supplementary Figure S12 *Model estimates of new nursing home placement odds ratio for each hospital against the number of physiotherapists per five stroke unit beds. Horizontal line represents an odds ratio of 1 for reference hospital 1. Vertical line represents the staffing levels of physiotherapists in hospital 1. Multivariable regression model was adjusted for pre-stroke mRS score, discharge mRS score, TACS, myocardial infarction or ischemic heart disease, complication, brain lateralization, and acute hospital length of stay, after multiple imputation for missing covariate data.*

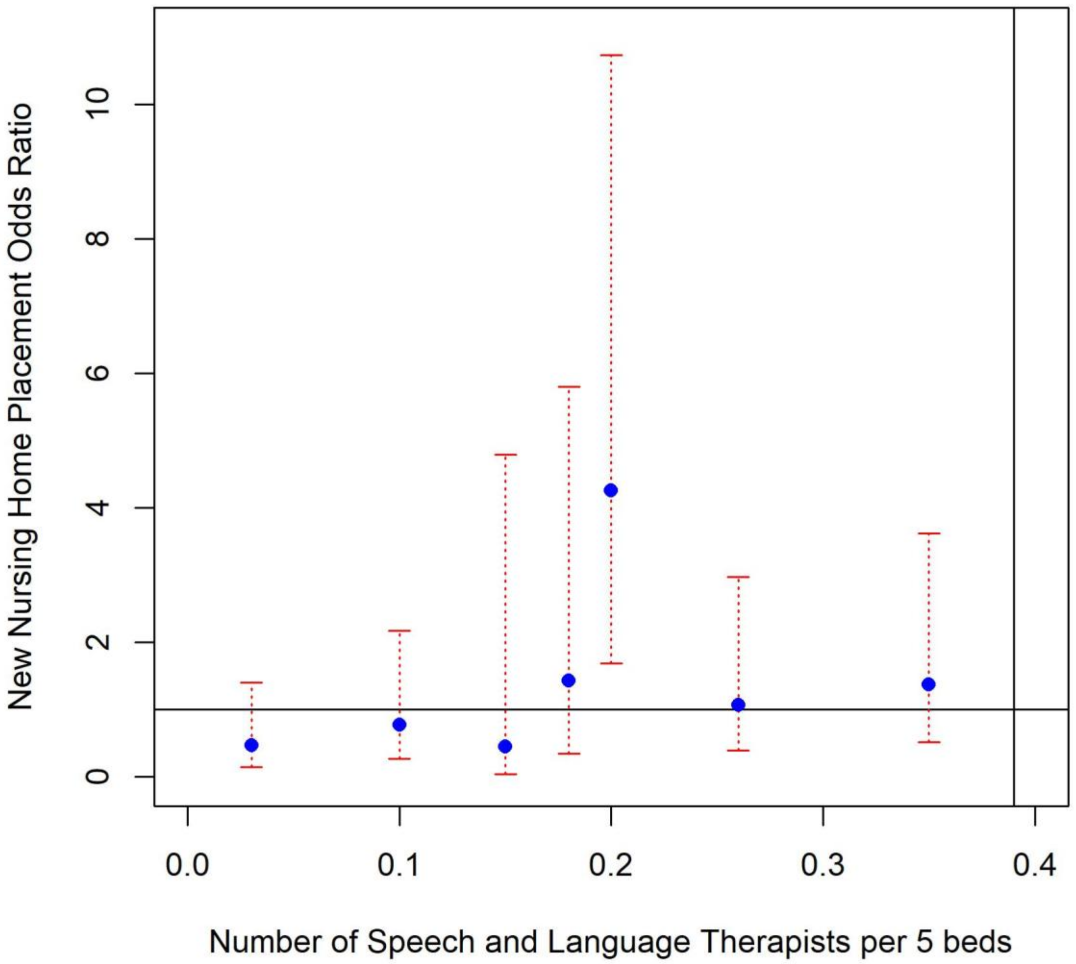

Supplementary Figure S13 *Model estimates of new nursing home placement odds ratio for each hospital against the number of speech and language therapists per five stroke unit beds. Horizontal line represents an odds ratio of 1 for reference hospital 1. Vertical line represents the staffing levels of speech and language therapists in hospital 1. Multivariable regression model was adjusted for pre-stroke mRS score, discharge mRS score, TACS, myocardial infarction or ischemic heart disease, complication, brain lateralization, and acute hospital length of stay, after multiple imputation for missing covariate data.*

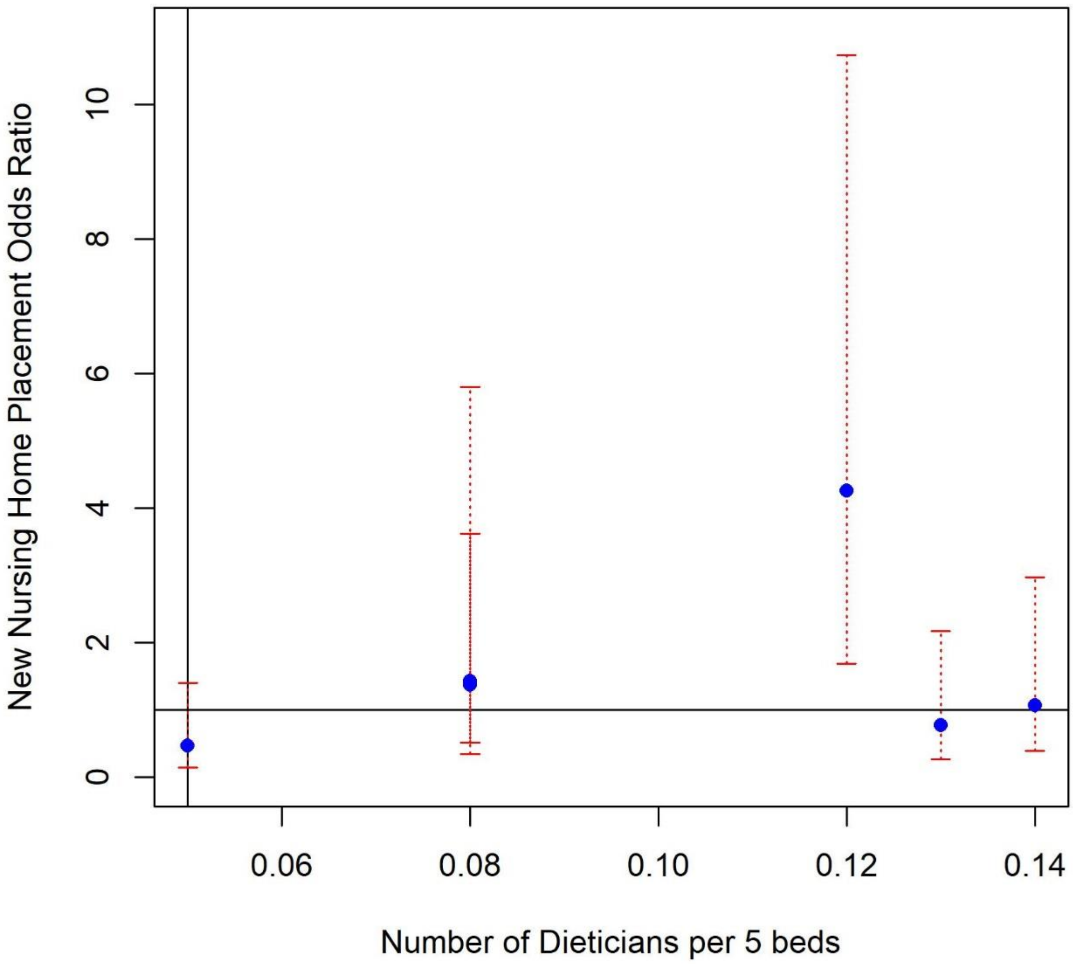

Supplementary Figure S14 *Model estimates of new nursing home placement odds ratio for each hospital against the number of dieticians per five stroke unit beds. Horizontal line represents an odds ratio of 1 for reference hospital 1. Vertical line represents the staffing levels of dieticians in hospital 1. Multivariable regression model was adjusted for pre-stroke mRS score, discharge mRS score, TACS, myocardial infarction or ischemic heart disease, complication, brain lateralization, and acute hospital length of stay, after multiple imputation for missing covariate data.*

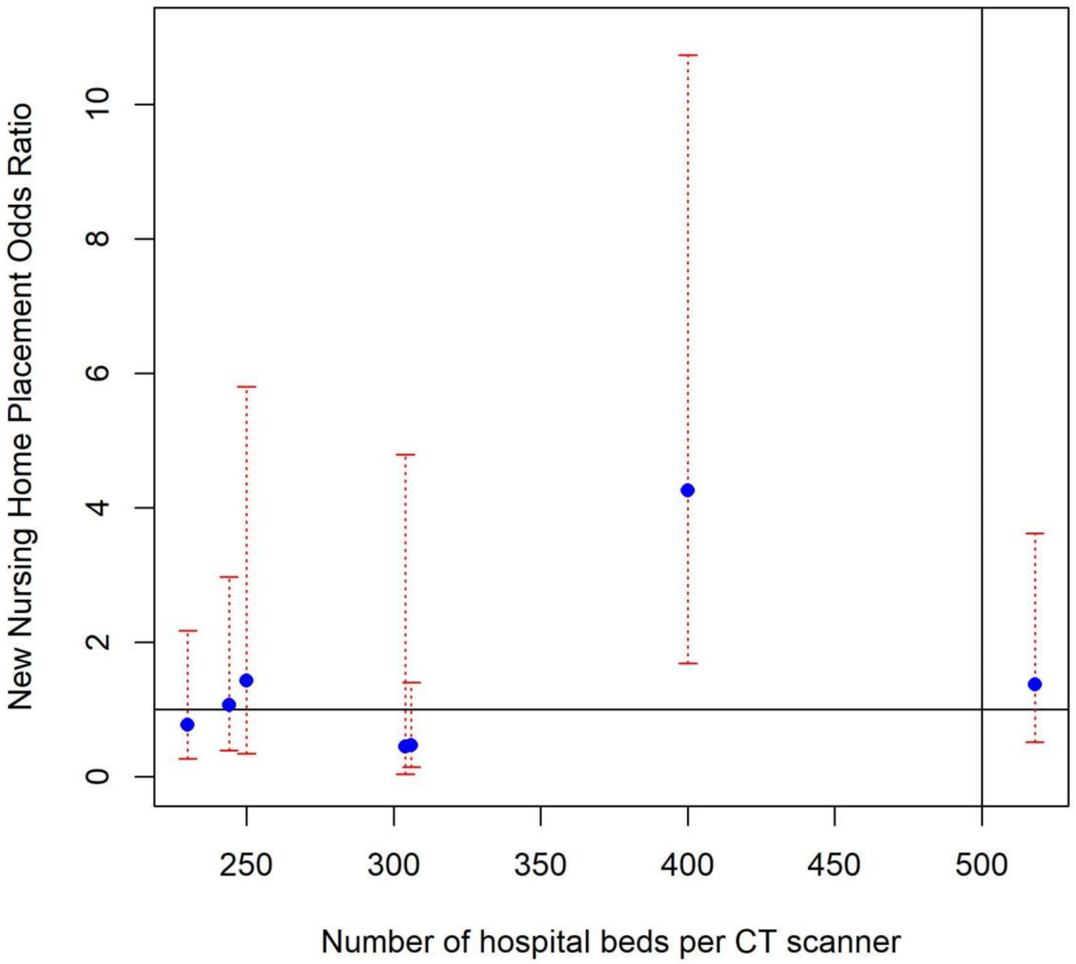

Supplementary Figure S15 *Model estimates of new nursing home placement odds ratio for each hospital against number of hospital beds per CT scanner. Horizontal line represents an odds ratio of 1 for reference hospital 1. Vertical line represents the numer of hospital beds per CT scanner in hospital 1. Multivariable regression model was adjusted for pre-stroke mRS score, discharge mRS score, TACS, myocardial infarction or ischemic heart disease, complication, brain lateralization, and acute hospital length of stay, after multiple imputation for missing covariate data.*

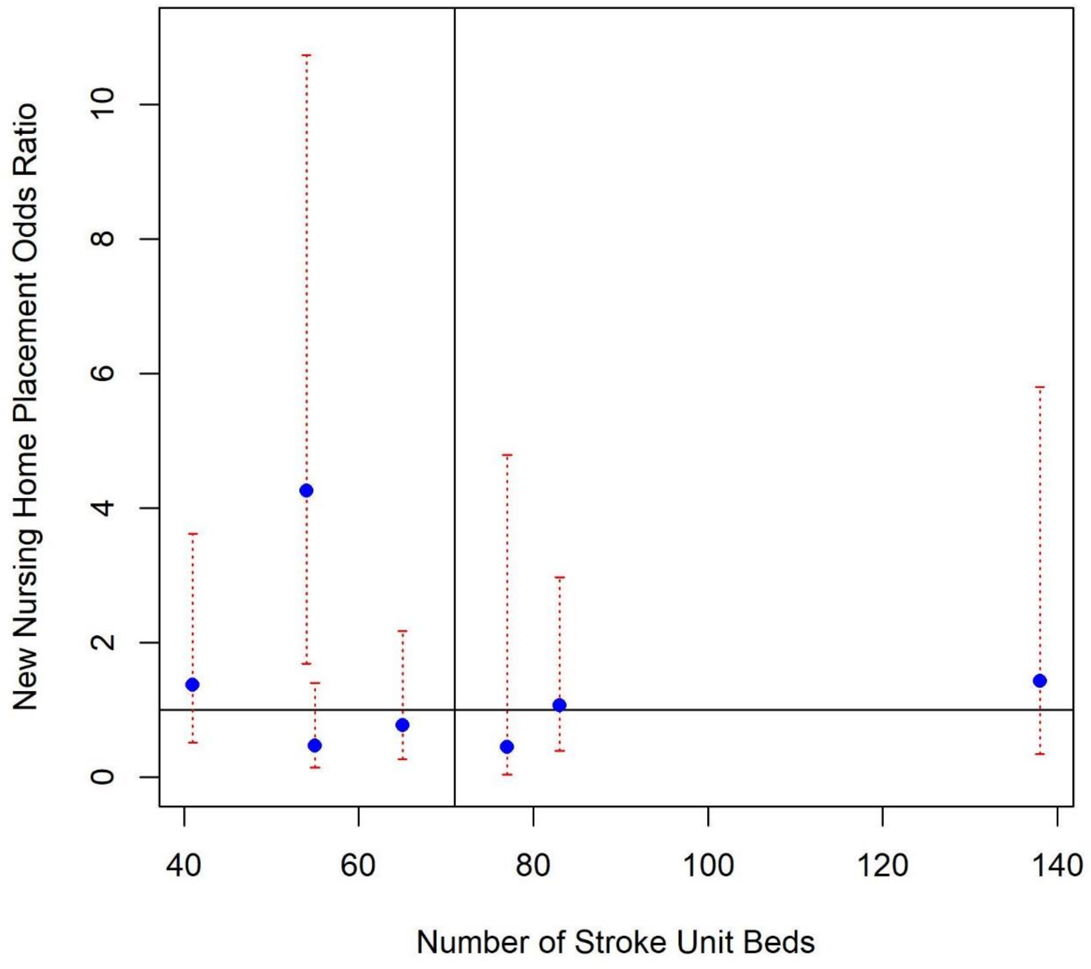

Supplementary Figure S16 *Model estimates of new nursing home placement odds ratio for each hospital against number of stroke unit beds per 100 admissions. Horizontal line represents an odds ratio of 1 for reference hospital 1. Verical line represents the number of stroke unit beds for hospital 1. Multivariable regression model was adjusted for pre-stroke mRS score, discharge mRS score, TACS, myocardial infarction or ischemic heart disease, complication, brain lateralization, and acute hospital length of stay, after multiple imputation for missing covariate data.*

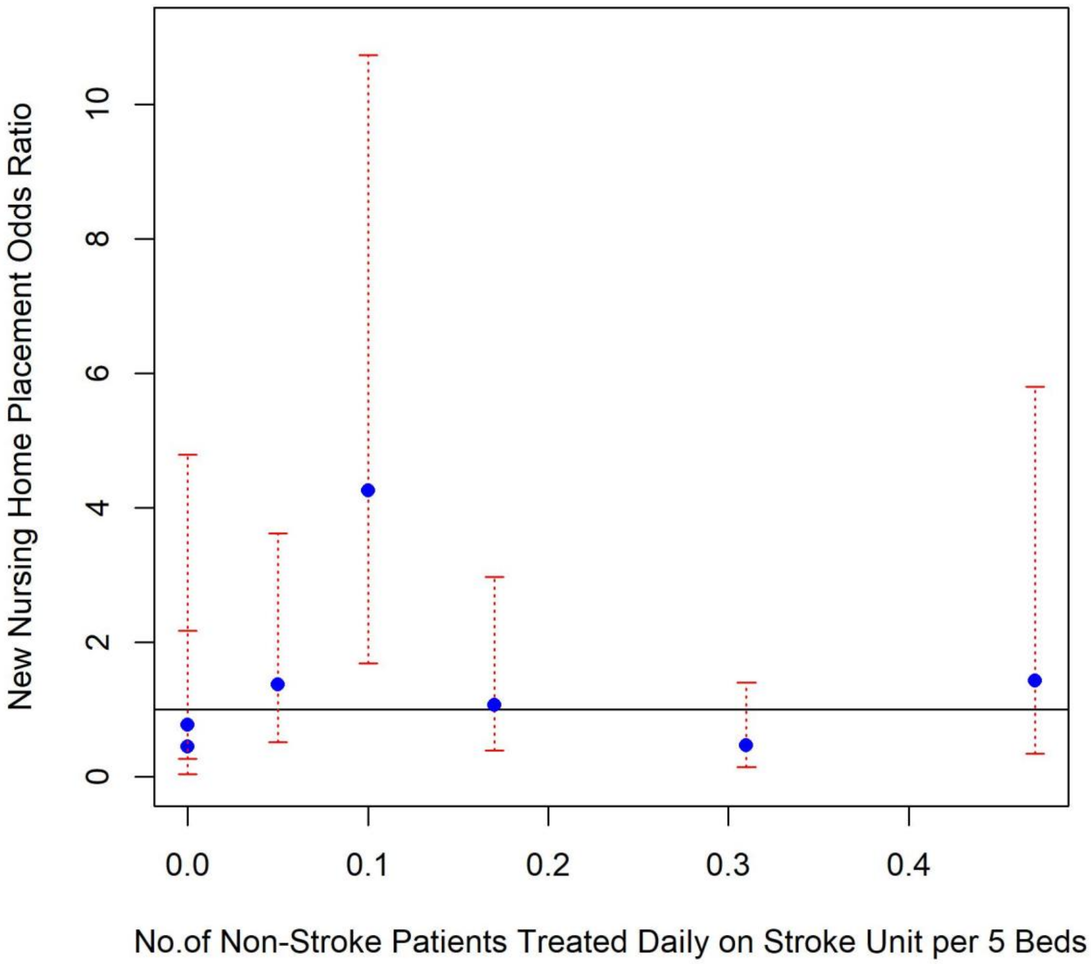

Supplementary Figure S17 *Model estimates of new nursing home placement odds ratio for each hospital against number of non-stroke patients treated daily on the stroke unit per five beds. Horizontal line represents an odds ratio of 1 for hospital 1. Verical line represents the number of non-stroke patients treated daily on the stroke unit of hospital 1. Multivariable regression model was adjusted for pre-stroke mRS score, discharge mRS score, TACS, myocardial infarction or ischemic heart disease, complication, brain lateralization, and acute hospital length of stay, after multiple imputation for missing covariate data.*

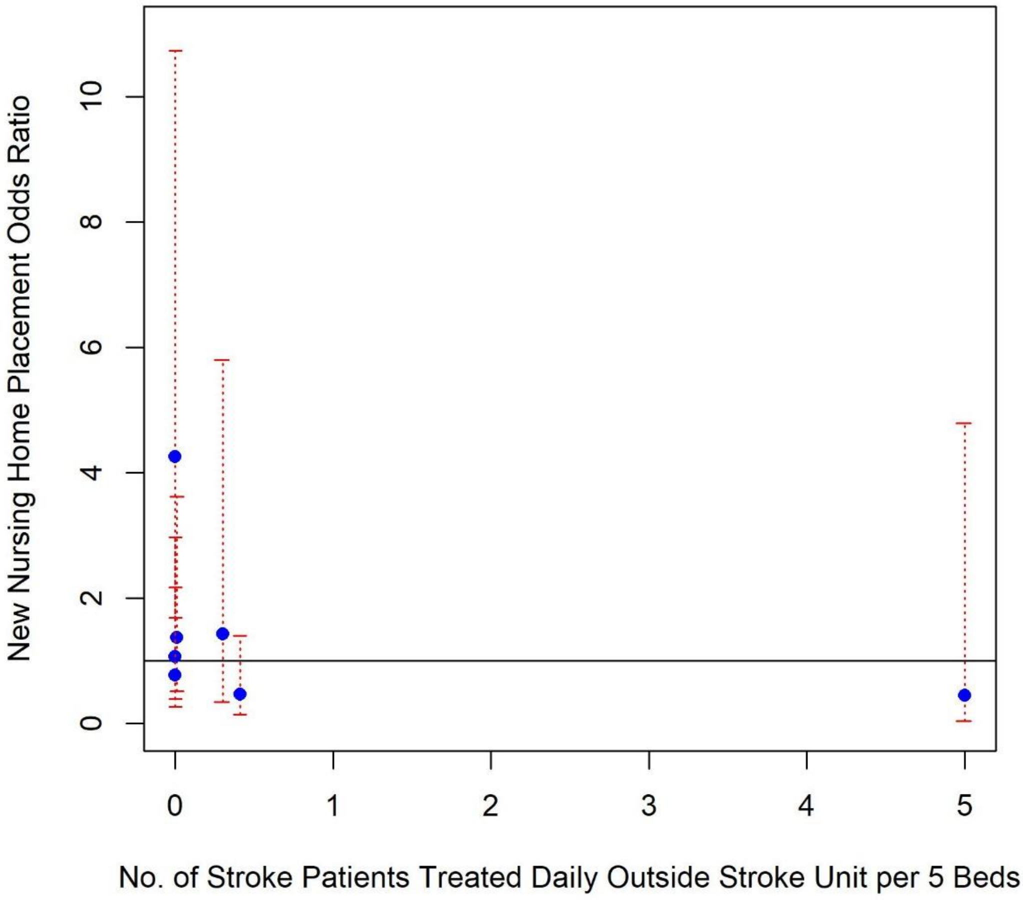

Supplementary Figure S18 *Model estimates of new nursing home placement odds ratio for each hospital against number of patients with stroke treated daily outside the stroke unit per five beds. Horizontal line represents an odds ratio of 1 for reference hospital 1. Verical line represents the number of patients with stroke treated outside the stroke unit per five beds for hospital 1. Multivariable regression model was adjusted for pre-stroke mRS score, discharge mRS score, TACS, myocardial infarction or ischemic heart disease, complication, brain lateralization, and acute hospital length of stay, after multiple imputation for missing covariate data.*

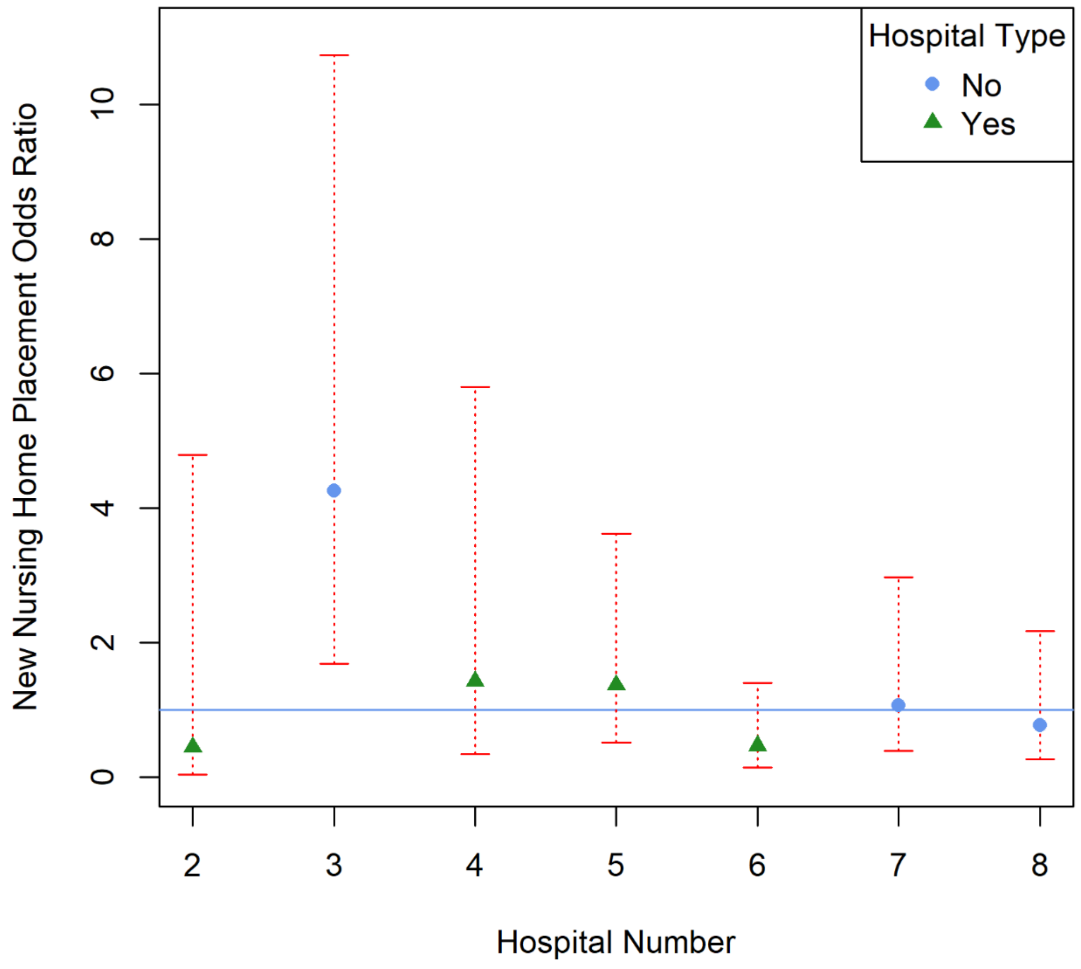

Supplementary Figure S19 *Model estimates of new nursing home placement odds ratio for each hospital against presence of early supported discharge (ESD) policy. Horizontal line represents an odds ratio of 1 for reference hospital 1; its blue colour represents that it does not have an ESD policy. Multivariable regression model was adjusted for pre-stroke mRS score, discharge mRS score, TACS, myocardial infarction or ischemic heart disease, complication, brain lateralization, and acute hospital length of stay, after multiple imputation for missing covariate data.*

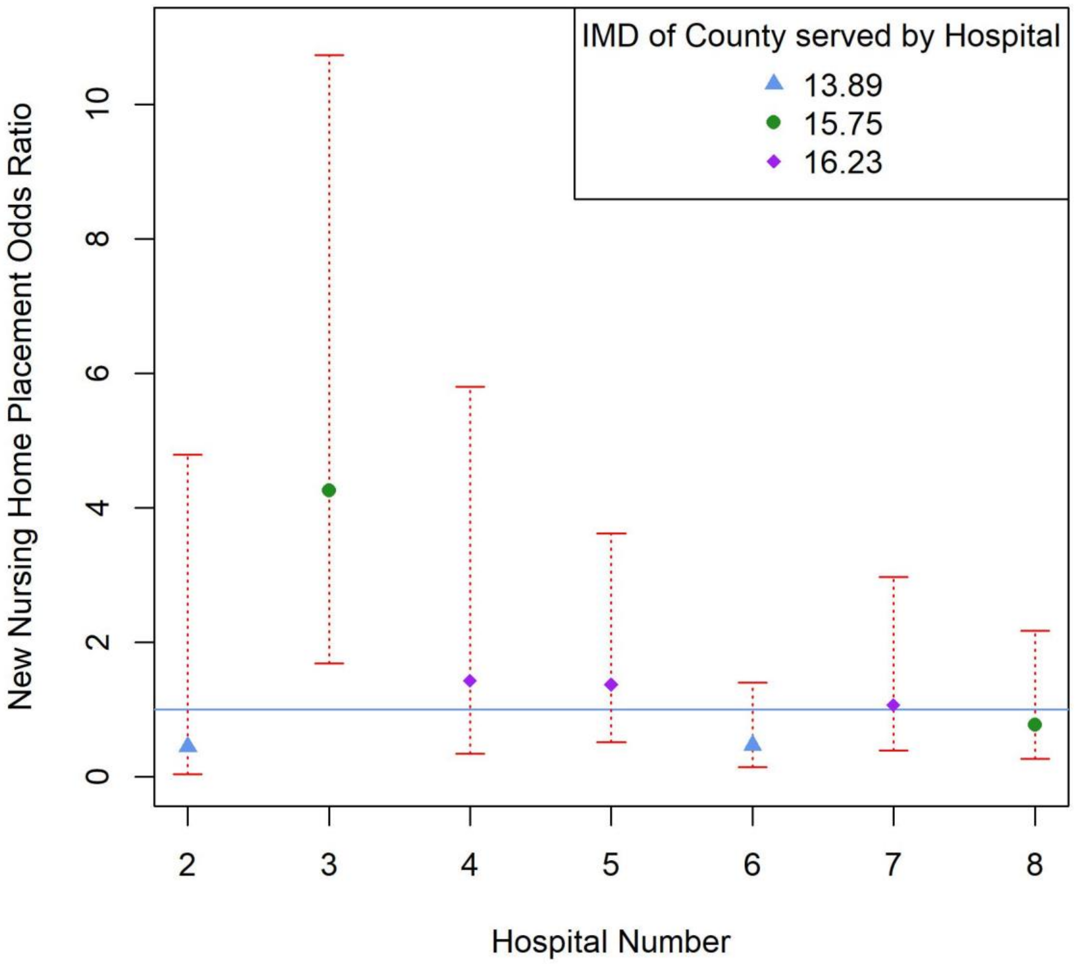

Supplementary Figure S20 *Model estimates of new nursing home placement odds ratio for each hospital against mean Index of Multiple Deprivation (IMD) score of the counties in which the hospital services, with 95% confidence intervals. Horizontal line represents an odds ratio of 1 for reference hospital 1; its blue colour represents that hospital 1 is located in a county with an IMD mean score of 13.89. Multivariable regression model was adjusted for pre-stroke mRS score, discharge mRS score, TACS, myocardial infarction or ischemic heart disease, complication, brain lateralization, and acute hospital length of stay, after multiple imputation for missing covariate data.*
